# A new screening tool for early recognition of ATTRv polyneuropathy in clinical practice: AmyloScan^®^

**DOI:** 10.1007/s00415-025-13338-z

**Published:** 2025-09-04

**Authors:** Juliane Sachau, Lena Rhode, Maike F. Dohrn, Jan Vollert, Stefanie Rehm, Manon Sendel, Katrin Hahn, Timon Hansen, Stefan Gingele, Thomas Skripuletz, Klarissa Stürner, Elena Enax-Krumova, Ralf Baron

**Affiliations:** 1 Division of Neurological Pain Research and Therapy, Department of Neurology, University Hospital Schleswig-Holstein, Campus Kiel, Arnold-Heller-Str. 3, Haus D, 24105 Kiel, Germany; 2 Department of Neurology, Medical Faculty of the RWTH Aachen University, Aachen, Germany; 3 Scientific Center for Neuropathic Pain, Medical Faculty of the RWTH Aachen University, Aachen, Germany; 4 Department of Clinical and Biomedical Sciences, Faculty of Health and Life Sciences, University of Exeter, Exeter, UK; 5 Department of Neurology, Charité-Universitätsmedizin Berlin, Corporate Member of Freie Universität Berlin, Humboldt-Universität Zu Berlin, Berlin Institute of Health (BIH), Berlin, Germany; 6 Berlin Institute of Health at Charité (BIH)—Universitätsmedizin Berlin, Charitéplatz 1, 10117 Berlin, Germany; 7 Onkologicum HOPA, Hamburg, Germany; 8 Department of Neurology, Hannover Medical School, Hanover, Germany; 9 Hannover Medical School, Amyloidosis Centre Lower Saxony, Hannover, Germany; 10 Department for Neurology, University Hospital Schleswig-Holstein, Campus Kiel, Kiel, Germany; 11 Department of Neurology, BG University Hospital Bergmannsheil GmbH, Ruhr-University Bochum, Bochum, Germany

**Keywords:** Hereditary transthyretin amyloidosis, Red flags, Bedside sensory test, CIDP, TTR silencers, TTR stabilizers

## Abstract

Hereditary transthyretin (ATTRv) amyloidosis is a progressive multisystem disorder, mainly characterized by cardiac dysfunction and polyneuropathy. Due to its rarity and heterogeneous presentation, diagnosis is often delayed, which has a direct impact on the initiation of treatment and, therefore, span and quality of life. To facilitate early disease recognition, we aimed to develop and validate a new screening tool for early identification of ATTRv amyloidosis with polyneuropathy (AmyloScan^®^). Potential disease characteristics were identified via literature screening, interviews and detailed examination of ATTRv amyloidosis patients (*n* = 10) and controls (CIDP: chronic inflammatory demyelinating polyneuropathy, *n* = 15; diabetic polyneuropathy, *n* = 15) using validated questionnaires, and autonomic and sensory testing. The preliminary AmyloScan® was developed and validated in patients with polyneuropathy caused by ATTRv amyloidosis (*n* = 21), CIDP (*n* = 19), or others (*n* = 43). By sensitivity and discriminant analyses, we determined the most characteristic item combinations for ATTRv amyloidosis. Cut-off values were defined by Receiver Operator Curves. The final AmyloScan® includes 12 questions and two sensory bedside tests. All items require simple yes/no answers, resulting in a total score of 0–14 points and two cut-off values: A value of ≥ 4 indicates an increased chance (sensitivity: 95.2%, specificity: 72.6%); a value of ≥ 6 indicates a high chance of ATTRv polyneuropathy (sensitivity: 81.0%, specificity: 93.5%). The AmyloScan^®^ is a simple time- and cost-efficient screening tool with a high discriminative value. Regular use in clinical practice might enable an earlier diagnosis with impact on treatment considerations and quality of life.

**Supplementary Information:**

The online version contains supplementary material available at 10.1007/s00415-025-13338-z.

## Introduction

Hereditary transthyretin (ATTRv) amyloidosis is a rare, progressive, fatal disease caused by misfolded transthyretin (TTR) protein that accumulates as amyloid fibrils in multiple organs, particularly nerves and heart, but also in the eyes, the central nervous system, and kidneys [1]. The clinical picture is, therefore, very heterogeneous, with cardiomyopathy and length-dependent polyneuropathy as the main, but not the only manifestations. Polyneuropathies in general are classified according to their etiology that can be discerned in about 70% of cases, i.e., up to one-third of the cases remain “polyneuropathies of unknown origin” [2]. The mid-global prevalence of ATTRv polyneuropathy is estimated to be 10,186 persons (range: 5,526–38,468) [3], but is likely to be even higher as only a minority of symptomatic ATTRv amyloidosis patients have been correctly diagnosed so far. In non-endemic regions, the estimated diagnostic delay is 3 to 4 years, with irreversible consequences on disease severity and quality of life [1]. This might be due to the fact that the initial manifestation of ATTRv amyloidosis, particularly in non-endemic areas, can occur even in older age without positive family history. Therefore, it is likely that at least some patients diagnosed with “polyneuropathy of unknown origin” may actually have underlying ATTRv amyloidosis. The delayed diagnosis is in particular fatal because untreated, the median survival is reported to be about 7 years in non-endemic regions [4, 5]. As specific therapies can slow down or even halt disease progression [6–10], there is an urgent need for an easy-to-use screening tool to detect ATTRv amyloidosis at the earliest stage.

Neurological symptoms of ATTRv amyloidosis might be specific enough already at the beginning of the disease to open a window for early diagnosis. In endemic regions, ATTRv amyloidosis polyneuropathy is characterized by a small fiber neuropathy (disturbances of cold and warm perception, neuropathic pain) with early involvement of motoric disturbances that rapidly (within months) progresses to a sensorimotor neuropathy with disturbances of the sensory function for all qualities and paresis [1]. In non-endemic regions, however, the presentation of ATTRv polyneuropathy is diverse [11]. In general, large-fiber involvement and upper limb weakness can be present already at an early stage. Autonomic symptoms such as erectile dysfunction, diarrhea, and constipation as well as postural hypotension also typically occur early in the course of the disease.

The aim of this project was to develop a time- and cost-efficient questionnaire and a short sensory testing procedure based on the most characteristic pattern of neurological symptoms to identify patients with ATTRv polyneuropathy and to distinguish them from polyneuropathies of other origin, in particular, the most frequent misdiagnosis, CIDP (chronic inflammatory demyelinating polyneuropathy).

## Methods

**Fig. 1:**
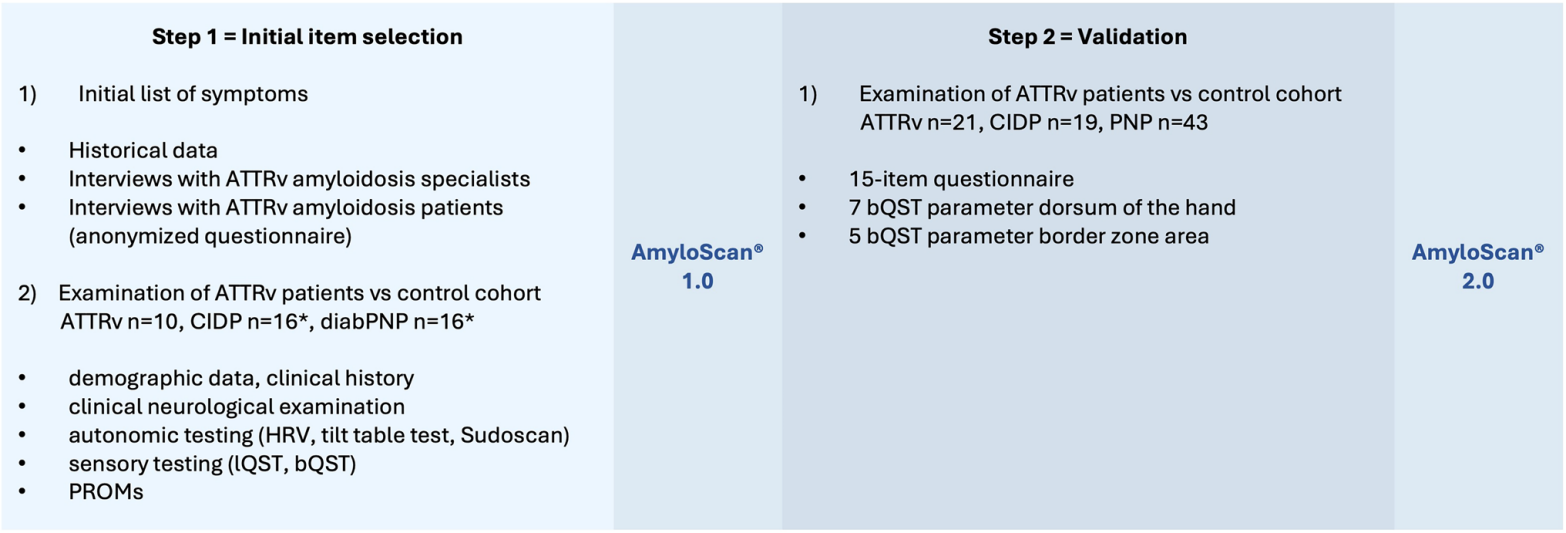
Study schedule. ATTRv amyloidosis, hereditary transthyretin amyloidosis; CIDP, chronic inflammatory demyelinating polyneuropathy; PNP, polyneuropathy; diabPNP, diabetic polyneuropathy; HRV, heart rate variability; lQST, laboratory quantitative sensory testing; bQST, bedside quantitative sensory testing; PROMs, patient-reported outcome measures. *one drop out per group

This study was conceptualized in two steps (Fig. 1): The first step was to select items characteristic for ATTRv amyloidosis from the literature, interviews or own collected data in order to develop a first version of the screening tool (AmyloScan^®^ 1.0.). The second step was to validate this first version and establish the final AmyloScan^®^.

The study protocol was approved by the local ethical committee of the Medical Faculty, Christian-Albrechts-University Kiel and conducted according to the Declaration of Helsinki. All participating patients gave their written informed consent prior to enrollment. The study protocol was registered in the German Clinical Trials Register (DRKS) (ID: DRKS00021577).

### ***Initial item selection for the AmyloScan***^***®***^

To meet the criteria of content validity, an initial list of characteristic clinical symptoms was first collected from a database including the history data of German patients who were assessed in the Division of Neurological Pain Research and Therapy in Kiel and secondly from structured interviews of 15 patients attending regular patient meetings using an anonymized questionnaire and from exchange with amyloidosis specialists. The structured patient interview included open questions on symptoms at the time of the initial manifestation and during the course of the disease (Supplement Table 1). Other potentially relevant items, including specific pain descriptors, were also included. Patients were asked to answer these questions in as much detail as possible and in their own words. A screening of the literature was performed to identify further etiology-specific items.

In a second step, we carefully examined 10 patients with ATTRv amyloidosis treated in two centers (University Hospital Kiel and Aachen) and compared them with a control cohort from Kiel consisting of 16 patients with CIDP and 16 patients with diabetic polyneuropathy (diabPNP). All patients were assessed by the same two examiners (an experienced neurologist and a student trained by the same neurologist). The assessment included demographic data (age, sex, ethnic) and clinical history (e.g., disease course, symptoms at the beginning and during the course of the disease, comorbidities, family history), a clinical neurological examination, autonomic testing, and both laboratory quantitative sensory testing (lQST) and bedside sensory testing (bQST). Several validated questionnaires were used to assess pain intensity and pain quality (Neuropathic Pain Symptom Inventory, NPSI; PainPREDICT; painDETECT questionnaire), quality of life (SF-36), and autonomic dysfunction (Compound Autonomic Dysfunction Test; CADT). In order to avoid an influence of medication on the test results and thus reflect the greatest symptom burden, the CIDP patients were examined shortly before the next administration of medication (IVIG, cortisone).

#### Inclusion criteria

Only patients suffering from one of the above-mentioned disease etiologies, i.e., ATTRv polyneuropathy, CIDP, or diabPNP were included. Medications were not exclusion criteria but were documented in detail. Exclusion criteria were as follows: pain syndromes of other causes as well as other relevant comorbidities potentially confounding the test results, e.g., severe psychiatric diseases.

#### Autonomic testing

Autonomic dysfunction was assessed in a standardized way with the heart rate variability test, the tilt table test and the Sudomotor test. Heart rate variability was assessed while lying down at rest and during deep breathing by use of computer-assisted ECG recording (ProSciCard III, MediSyst GmbH). Orthostatic dysregulation was tested using the tilt table test with continuous blood pressure and heart rate measuring while lying down, 1, 3, 5, 10 min after orthostasis and 1 min after the end of orthostasis (Finapres Medical System B.V. (Finapres^®^ NOVA). Sudomotor function was tested using the Sudoscan, a non-invasive device for measuring electrochemical skin conductivity [12].

#### Laboratory and bedside quantitative sensory testing

Laboratory QST (lQST) was performed according to the standardized protocol of the German Research Network on Neuropathic Pain (DFNS), which uses different calibrated stimuli to detect positive and negative sensory signs [13]. The lQST battery consists of seven tests measuring 13 thermal and mechanical parameters. Briefly, thermal detection thresholds for the perception of cold (CDT) and warm (WDT), thermal pain thresholds for cold (CPT) and heat (HPT), thermal sensory limen (TSL), paradoxical heat sensation (PHS), mechanical detection thresholds for touch (MDT) and vibration (VDT), mechanical pain threshold (MPT), a stimulus–response-function for pinprick sensitivity (MPS), dynamic mechanical allodynia (DMA), pain summation to repetitive pinprick stimuli (wind-up ratio; WUR), and blunt pressure pain threshold (PPT) were assessed. For all QST values (except for DMA and PHS), z-scores were calculated that enable direct comparison with a reference database of age-, sex- and location-matched healthy subjects. Z-scores of zero represent the mean of healthy controls, z-scores above “0” indicate a gain of function (hyperalgesia), and z-scores below “0” indicate a loss of function (hypoesthesia, hypoalgesia). Z-scores outside the 95% confidence interval were defined as abnormal loss/gain of function. DMA and PHS were given with original values since both phenomena are absent under physiological conditions (DMA: 0–100 numeric rating scale; PHS: numbers of PHS, 0–3).

A previously developed bedside sensory testing battery (bQST) was used as a cost-effective and easy-to-conduct alternative to the lQST [14, 15]. Several mechanical and thermal stimuli were applied to the skin. Patients had to rate 1) whether the stimulus was perceived/not perceived or painful/not painful (yes/no) and 2) the perception or pain intensity of each stimulus using an 11-point numerical rating scale (NRS; 0 = no perception/no pain, 10 = strongest imaginable perception/strongest imaginable pain). The complete protocol is described elsewhere [14, 15]. Briefly, thermal pain/perception was investigated using four metal pieces/cubes with a defined temperature (8 °C, 22 °C, 37 °C, 45 °C), and dynamic and static touch sensation were examined using a Q-tip, a 64-mN von Frey hair, a Neuropen filament (monofilament with defined pressure of 10 g), and a 64-mN von Frey hair. Pinprick hyperalgesia and hypoalgesia as well as temporal pain summation were detected using a 0.7 mm CMS hair and a Neuropen with a Neurotip (disposable needle with a defined pressure of 40 g). For dynamic mechanical allodynia and postallodynia sensation pain a Q-tip was brushed over the skin. A bedside algometer (10-mL syringe sealed with a plug and felt pad with a contact area of 1 cm^2^) was placed above a muscle to evaluate deep somatosensory pressure pain. Finally, the vibration detection threshold was investigated using a standardized tuning fork (64 Hz, 8-point scale).

Both lQST and bQST were performed in affected areas of the upper and lower limbs. On the upper limb, the back of one hand (in the case of a known CTS: in the supply area of the ulnar nerve) and, on the lower limb, the dorsum of one foot were used. Since ATTRv polyneuropathy is rapidly progressing from distal to proximal, patients, who were tested late in the course of the disease would have been expected to have an exclusive loss of function, especially in the foot. To detect possible earlier somatosensory abnormalities in particular, a border zone area was determined, defined as the most proximal area that is still clinically affected.

Based on the data from step 1, two neurologists experienced in amyloidosis management and clinical research, compiled a list of 15 questions representative of several clinical dimensions of ATTRv amyloidosis and 12 bedside sensory parameters (1st draft, AmyloScan^®^ 1.0, Supplement Table 3).

### Validation

The initial 27-item version of AmyloScan^®^ 1.0 (15 questions and 12 bedside parameters) was tested in a cohort of 83 patients with polyneuropathy due to ATTRv amyloidosis, CIDP, or other causes. The aim was to identify a cut-off score with the best discriminative value for distinguishing between ATTRv polyneuropathy and polyneuropathies of other cause. All patients were examined in the centers by pain specialists with experience in pain management or an adequately trained prospective physician, not-blinded for the diagnosis.

21 genetically confirmed patients with ATTRv amyloidosis (10 male and 11 female) and control patients who suffered either from other polyneuropathies of different etiology (CIDP (*n* = 19) or PNP (*n* = 43), established by a clinical neurologist according to the current diagnostic guidelines) were tested at the Department of Neurology, Christian-Albrechts-University, Kiel, Germany or, in case of longer distance to the clinic and/or limited mobility, at home.

### Statistical methods and assessment of the psychometric properties

For the initial item selection (step 1), all collected data were analyzed for possible significant differences between ATTRv amyloidosis and CIDP or diabPNP. Differences in dichotomized variables were calculated using the chi-square test (or Fisher's exact test for *n* < 5). Differences in metric variables were calculated using the Mann–Whitney *U* test. *P*-values < 0.05 were defined as significant.

Variables that showed significant (*p* < 0.05) differences between ATTRv amyloidosis and controls and those that were still considered clinically relevant despite lack of significance (see results section) were used for further analysis (step 2). If two items were derived from the same variable, only the item with higher significance was carried forward. For the final AmyloScan^®^ only variables with (1) statistical significance, (2) clinical plausibility, and (3) ease of applicability were selected. Thus, one variable was simplified post hoc (dichotomous variable “rapid worsening yes/no” instead of four item scale “improvement—no change—slow worsening—rapid worsening”).

A discriminant analysis was carried out with the remaining variables. As sensitivity analysis, a Leave-one-out (LOO) analysis was conducted, and accuracy was compared between full and LOO results. Based on the discriminant function, a simplified function using only the included yes/no items in an additive score was created to enable simple application and increase external validity. For both functions, Receiver Operator Curves (ROC) were plotted, and the areas under the curve (ROC AUC) were calculated, along with bootstrapping based 95% confidence intervals. In case of overlapping confidence intervals, we preferred the simple function due to its practical advantages and likely higher external validity, as simple scores as less likely to result in overfitting. Finally, the Youden index (Sensitivity + Specificity—1) was calculated for each value of the chosen function to determine the optimal cut-off value for the total score of the AmyloScan®. The internal consistency of the questionnaire was assessed by calculating Cronbach’s alpha coefficient.

## Results

### Step 1

Upon retrospective questionnaire assessment, nearly all ATTRv amyloidosis patients (*n* = 15) described polyneuropathy symptoms (93.3%), most frequently sensory dysfunction (80%), as first manifestation. Interestingly, more than half of the patients (53.3%) additionally reported muscle weakness, particularly in the lower limbs, as first symptom. During the course of the disease, additional symptoms occurred, i.e., further sensory deficits in particular hypoesthesia (73.3%), tingling (66.7%), and pain (46.7%); the latter was described as burning by almost all patients (85.7%), and motor deficits like weakness (60.0%). Signs of autonomic neuropathy were reported by most of the patients (86.7%), especially gastrointestinal complaints, weight loss, and dizziness. Nearly all patients (93.3%) reported a heterogeneous picture of cardiac symptoms during the course of the disease.

Data from the detailed examination of 10 patients with ATTRv amyloidosis polyneuropathy (7 male, 3 female), 15 patients with CIDP (8 male, 7 female; one not analyzed due to competing alcohol abuse) and 15 patients with diabPNP (12 male, 3 female; one not analyzed due to not clearly confirmed polyneuropathy) were analyzed. One patient with diabPNP and one patient with CIDP were excluded from the analysis due to possible competing diseases (alcohol abuse). In ATTRv amyloidosis the most common gene mutation was Val30Met, most patients were examined in an early disease stage with preserved walking ability and only mild sensory neuropathy, i.e., Coutinho stage 1 (70%) or PND score 1 (50%), whereas no one was wheelchair bound or bedridden. All patients with ATTRv amyloidosis were treated with a disease-modifying drug (patisiran or tafamidis). Characteristics of ATTRv amyloidosis patients are shown in Supplement Table 2.

**Fig. 2:**
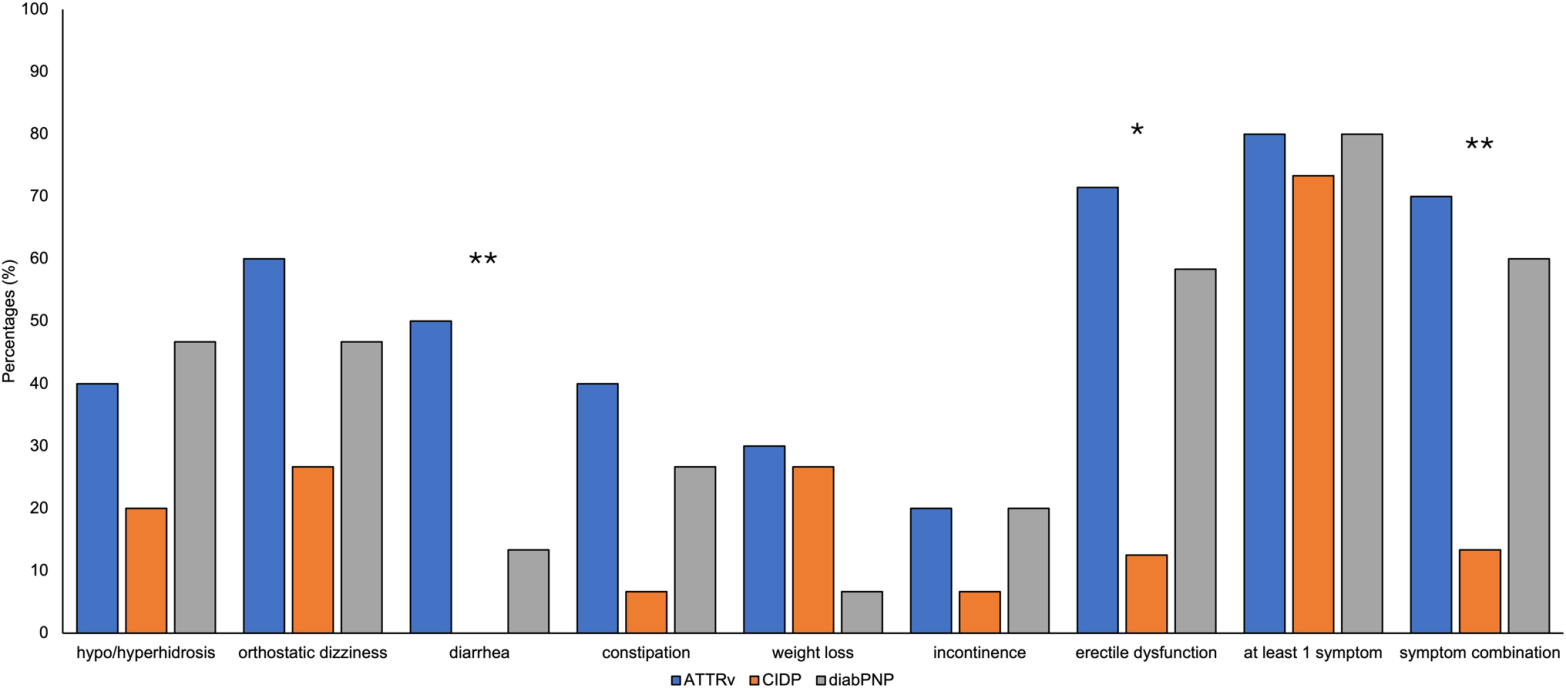
Frequencies of autonomic symptoms in ATTRv amyloidosis (n = 10) vs. CIDP (*n* = 15) and diabPNP (*n* = 15). * ATTRv vs. CIDP (*p < 0.05; **p < 0.01)

ATTRv amyloidosis patients and CIDP patients were similar in sex, age and BMI, whereas patients with diabPNP were significant older (58.5 vs. 69.3 years; *p* = 0.012) and showed a higher BMI (25.3 vs. 30.9; *p* = 0.026). While the symptom duration and intake of pain drugs was similar between ATTRv amyloidosis and both control groups, patients with ATTRv amyloidosis most commonly described their symptoms as being stable (55.6%) or progressive (44.4%) during the current treatment. In contrast, around three quarters of CIDP and diabPNP patients reported a progressive course of the disease. While there were no differences in the type of initial symptoms (mostly or even exclusively neurological symptoms), patients with ATTRv amyloidosis also described symptoms during the disease indicative of other organ manifestation, i.e., cardiac, ocular, cerebral, or other (cough, fatigue, dysphagia). A history or current symptoms of a carpal tunnel syndrome (CTS) were present in most of the patients with ATTRv amyloidosis (70%), while it was only reported by a minority of patients with CIDP (13.3%; *p* = 0.009) and diabPNP (26.7%; *p* = 0.049). Symptoms of restless legs syndrome were more frequently reported by ATTRv amyloidosis patients compared with CIDP patients (33.3% vs. 0%; *p* = 0.042). While ATTRv amyloidosis and diabPNP did not differ significantly with regard to autonomic symptoms, autonomic dysfunctions, in particular diarrhea and erectile dysfunction and a combination of different symptoms, were more frequently described by ATTRv amyloidosis patients compared with CIDP patients (Fig. 2).

ATTRv amyloidosis presented similarly to CIDP and diabPNP on clinical neurological examination, particularly in terms of sensory symptoms. However, patients with ATTRv amyloidosis were characterized more frequently by weakness of the upper and lower limbs compared with both CIDP (upper limb: 60% vs. 6.7%; *p* = 0.007) and diabPNP (upper/lower limb: 60% vs. 6.7%; *p* = 0.007) as well as muscle atrophy (ATTRv: one patient only thenar muscle, three patients only thigh and/or lower leg, three patients generalized, i.e., both upper limb (thenar) and lower limb) and gait disturbances compared with diabPNP (70% vs. 6.7%; *p* = 0.002 and 70% vs. 20%; *p* = 0.034).

Although no significant differences were found between ATTRv amyloidosis and both control groups in terms of pain quality, the painDETECT questionnaire (PD-Q) profiles suggested a greater expression of burning and tingling in ATTRv amyloidosis patients. Overall, results must be interpreted with caution, since questionnaire results of only seven patients with ATTRv amyloidosis were available (missing data due to language barrier, death, and disease-related emotional stress).

#### Differences from sensory testing

Results of bQST at the dorsum of the hand revealed some differences between patients with ATTRv amyloidosis and both CIDP and diabPNP. Comparison of interval-scaled parameters (perception/pain intensity: NRS) indicated a higher pressure pain sensitivity in ATTRv amyloidosis versus CIDP patients (3.3 ± 0.42 vs. 2.2 ± 0.24; *p* = 0.006) and a lower sensitivity to vibration in ATTRv amyloidosis versus diabPNP patients (5.17 ± 0.51 vs. 7.23 ± 0.26; *p* = 0.002). Comparison of dichotomized bQST parameters (perception/pain: yes/no) indicated a more pronounced hypoesthesia to heat and cold in ATTRv amyloidosis versus CIDP patients, i.e., the 37 °C metal piece was less frequently perceived as warm (50% vs. 100%; p = 0.005) and the cooled side of the TipTherm less frequently perceived as cold (≤ 1 × perceived as cold: 33.3% in ATTRv vs. 0% in CIDP; *p* = 0.042). The lQST showed similar results, with ATTRv amyloidosis patients additionally showing abnormal pinprick hyperalgesia less frequently than diabPNP patients (11.1% vs. 60%; *p* = 0.033).

In keeping with the rapidly progressive distal-to-proximal nature of ATTRv amyloidosis, the lower leg was most commonly examined as the border zone area (70%), and the foot in only two patients. In contrast, the foot was defined as the border zone area in 46% and 60% of CIDP and diabPNP patients, respectively. Comparison of interval-scaled bQST parameters (perception/pain intensity: NRS) indicated a higher pressure pain sensitivity in ATTRv amyloidosis versus both CIDP (4.29 ± 0.62 vs. 2.82 ± 0.52; *p* = 0.042) and diabPNP patients (2.8 ± 0.40; *p* = 0.040). Comparison of dichotomized bQST parameters (perception/pain: yes/no) revealed cold hypoesthesia in ATTRv amyloidosis versus CIDP and diabPNP patients, i.e., the 22 °C metal piece was less frequently perceived as cold (50% vs. 93.3%; *p* = 0.023 (both)). Again, similar results were observed using lQST.

#### Differences upon autonomic testing

Tilt table, heart rate variability and Sudoscan tests revealed no significant differences between patients with ATTRv amyloidosis and both CIDP and diabPNP (data not shown here).

### Step 2

Although some items of step 1 did not reach statistical significance, they were nevertheless included in step 2 as some differences might have been lost due to the low number of patients (PD-Q items pain (i.e., burning) and tingling), imprecisely formulated questions (family history), and significance in the clinical examination but not the anamnesis (muscle atrophy and gait impairment). Consequently, some questions were reworded or added (i.e., misdiagnosis of CIDP in the past). In total, 21 patients with ATTRv amyloidosis (10 male, 11 female), 19 patients with CIDP (10 male, 9 female), and 43 patients with PNP (22 male, 21, female; diabetic *n* = 19, chemotherapy-induced *n* = 6, alcoholic *n* = 2, toxic *n* = 1, critical-illness *n* = 1, paraneoplastic *n* = 1, vitamin B12 deficiency *n* = 1, unknown *n* = 12) were included.

None of the patients indicated problems answering the questions. Results are shown in Table 1. While the duration of the disease-caused symptoms and the course of the disease over the last 6 months were similar between the three disease etiologies, patients with ATTRv amyloidosis more frequently reported rapid symptom progression compared with patients with PNP. Comorbidities were characteristic for ATTRv amyloidosis, i.e., patients with ATTRv amyloidosis more frequently reported a history or symptoms of carpal tunnel syndrome and cardiac dysfunction compared with both CIDP and PNP and ocular dysfunction compared with PNP. In addition, muscle atrophy and autonomic dysfunction were more frequent in ATTRv amyloidosis. Interestingly, neuropathic pain patterns were more frequently reported by CIDP patients compared with ATTRv amyloidosis. Neuropathic pain descriptors (in particular burning and tingling) did not differ between the three groups (data not shown). Finally, a positive family history with similar symptoms compared to the index patient was more frequent in ATTRv amyloidosis compared with CIDP and PNP.

**Table 1:** Questions selected for AmyloScan® 1.0 and respective p-values for comparison of ATTRv amyloidosis vs. CIDP and PNP

	ATTRv (n = 21)	CIDP (n = 19)	PNP (n = 43)	ATTRv vs. CIDP	ATTRv vs. PNP
1. Symptom duration (years)	5,12 ± 3.75	7.72 ± 5.19	7.79 ± 8.71	0.078	0.687
2. Rapidly progressive total course of the symptoms since the onset of the disease? * (yes)	5 (23.8)	2 (10.5)	1 (2.3)	0.412	**0.012**
3. Rapidly progressive course of the symptoms over the last 6 months? * (yes)	2 (9.5)	0	6 (14.0)	0.490	1.000
4. Carpal tunnel syndrome? (yes)	19 (90.5)	4 (21.1)	10 (23.3)	** < 0.001**	** < 0.001**
5. Cardiac dysfunction? (yes)	19 (90.5)	3 (15.8)	16 (37.2)	** < 0.001**	** < 0.001**
6. Ocular dysfunction? (yes)	11 (52.4)	5 (26.3)	6 (14.0)	0.093	**0.001**
7. Muscle atrophia? (yes)	16 (76.2)	8 (42.1)	8 (18.6)	**0.028**	** < 0.001**
8. Restless legs syndrome? (yes)	6 (28.6)	8 (42.1)	13 (30.2)	0.370	0.891
9. Uncertainty when walking? (yes)	15 (71.4)	16 (84.2)	32 (74.4)	0.457	0.799
10. Slow gait? (yes)	15 (71.4)	12 (63.2)	25 (58.1)	0.577	0.303
11a. Hypo-/Hyperhidrosis? (yes)	12 (57.1)	4 (21.1)	11 (25.6)	**0.049**	**0.013**
11b. Diarrhea? (yes)	10 (47.6)	1 (5.3)	4 (9.3)	**0.004**	**0.001**
11c. Constipation? (yes)	9 (42.9)	2 (10.5)	11 (25.6)	**0.034**	0.162
11d. Nausea/Vomiting? (yes)	4 (19.0)	0	1 (2.3)	0.108	**0.036**
11e. Orthostatic dizziness? (yes)	12 (57.1)	11 (57.9)	17 (39.5)	0.962	0.184
11f. Weight loss? (yes)	10 (47.6)	2 (10.5)	10 (23.3)	**0.016**	**0.048**
11 g. Incontinence? (yes)	4 (19.0)	0	9 (20.9)	0.108	1.000
11 h. Erectile dysfunction? (yes)	5 (50.0)	1 (9.1)	13 (59.1)	0.063	0.631
12. Neuropathic pain? (yes)	11 (52.4)	16 (84.2)	24 (55.8)	**0.046**	0.796
12a. Neuropathic pain intensity (mean)	6.27 ± 1.40	6.64 ± 1.37	5.81 ± 1.69	0.507	0.517
12b. Neuropathic pain course? Stable Improving Worsening	3 (27.3) 2 (18.2) 6 (54.4)	3 (18.8) 1 (6.3) 12 (75.0)	3 (11.5) 2 (7.7) 21 (80.8)	0.483	0.260
13. Tingling (yes)	15 (71.4)	17 (89.5)	29 (67.4)	0.241	0.747
13a. Tingling intensity	3.93 ± 3.86	4.75 ± 2.37	3.30 ± 3.13	0.496	0.409
14. Positive family history^#^ (yes)	17 (81.0)	7 (36.8)	9 (20.9)	**0.009**	** < 0.001**
15. (Mis-)Diagnosis CIDP (yes)	0	n.a	0	n.a	n.a
15a. Improvement of symptoms following CIDP therapy (yes)	0	16 (84.2)	0	n.a	n.a

Given are total numbers and frequencies for dichotomous items with p-values of a chi-square test or Fisher’s exact test and mean values ± standard deviations for interval-scaled items with p-values of a Mann–Whitney *U* test. significant *p*-values are marked in bold

*n.a.* not applicable

*Original response options were dichotomized

^#^Depending on the disease, positive family history of PNP/CIDP/ATTRv amyloidosis

**Fig. 3:**
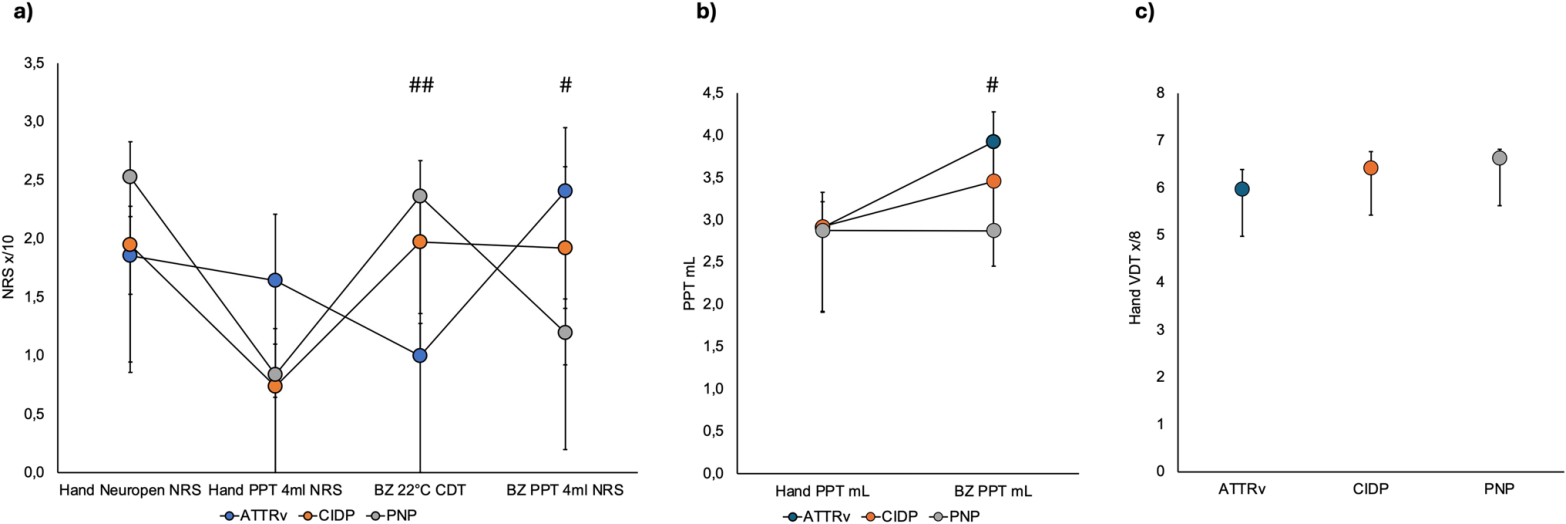
Profiles with selected bQST items of patients with ATTRv amyloidosis (n = 21) versus CIDP (*n* = 19) and PNP (*n* = 43) a) bQST parameters that were assessed using a 0–10 numerical rating scale (NRS), b) pressure pain threshold (PPT) in mL, c) vibration detection threshold (VDT) in x/8. Shown are the mean values and the standard error of the mean. BZ = border zone area. # ATTRv vs. PNP (#*p* < 0.05; ##*p* < 0.01)

**Fig. 4:**
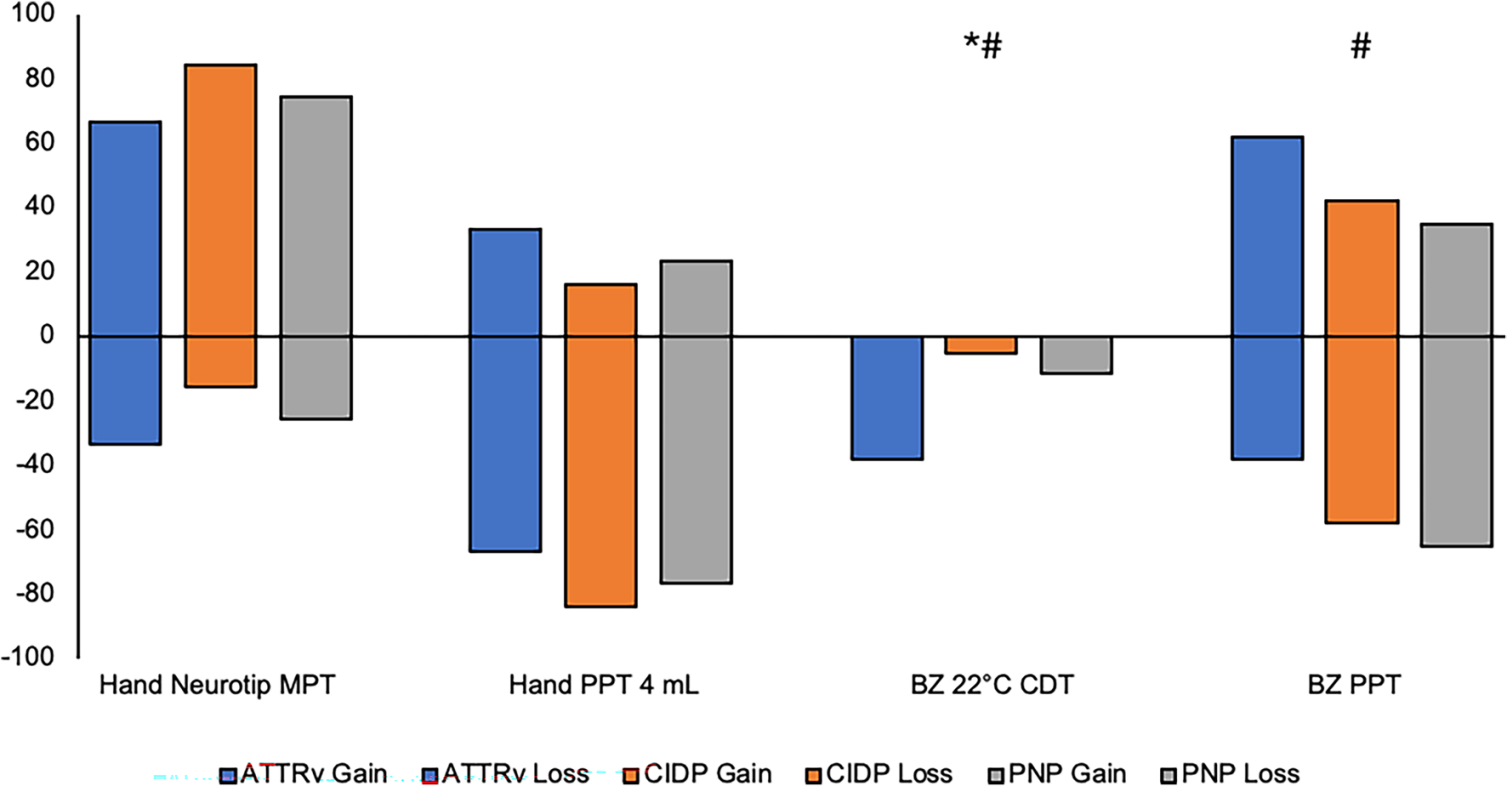
Frequencies of selected dichotomized bQST parameters (perception/pain: yes/no) of patients with ATTRv amyloidosis (*n* = 21) versus CIDP (*n* = 19) and PNP (*n* = 43). Shown are the frequencies of patients with gain of function (positive values, i.e., stimulus perceived/painful) and loss of function (negative values, i.e., stimulus not perceived/not painful). *MPT* mechanical pain threshold. *PPT* pressure pain threshold. *CDT* cold detection threshold. *BZ* border zone area. * ATTRv vs. CIDP (*p < 0.05); # ATTRv vs. PNP (#*p* < 0.05)

Comparison of the selected bQST items revealed statistical significances only for the border zone area. Patients with ATTRv amyloidosis were characterized by a cold hypoesthesia as assessed by cold intensity to a 22 °C metal cube and pressure pain hyperalgesia as assessed by both pain intensity to 4 mL pressure and pressure pain threshold in mL compared with patients with PNP (CDT: 1.0 ± 0.28 vs. 2.36 ± 0.31; *p* = 0.008; PPT 4 mL: 0.24 ± 0.54 vs. 1.20 ± 0.29; *p* = 0.034; PPT mL: 0.39 ± 0.35 vs. 0.29 ± 0.22; *p* = 0.013) (Fig. 3). Comparison of dichotomized bQST parameters also revealed a more frequent hypoesthesia to the 22 °C metal cube compared with CIDP (38.10% vs. 5.26%; *p* = 0.021) and diabPNP (11.63%; *p* = 0.013) and a more frequent pressure pain hyperalgesia to 4 mL compared with diabPNP (61.90% vs. 34.88%; *p* = 0.041) (Fig. 4).

Based on these results along with clinical relevance of questions and interdependence of items (e.g., multiple items derived from the same test), 14 items (12 questions, 2 bedside tests) were chosen for the final AmyloScan^®^ (see Table 2). Although the question regarding neuropathic pain resulted in a statistically significant difference between ATTRv amyloidosis and CIDP, it was excluded from the final analysis due to contradictory data compared to the literature. Some bQST items were excluded as they test similar sensory qualities and did not show any relevant improvement in the final AmyloScan®. In particular, the exclusion of the interval-scaled bQST items (border zone: 22°CDT NRS, border zone: PPT 4 mL NRS, border zone: PPT mL) allowed further simplification of the screening tool.

**Table 2:** Items of the AmyloScan^®^

Questions (yes/no)
1. Have your symptoms rapidly worsened/increased since they began?
2. Do you currently have/have you ever had carpal tunnel syndrome or symptoms of carpal tunnel syndrome (pain/dysesthesia from thumb to ring finger)?
3. Do you have heart problems such as breathlessness, palpitations, cardiac arrhythmia, or other pre-existing heart conditions?
4. Do you have visual problems such as blurred vision (not as part of presbyopia)
5. Do you suffer from muscle atrophy in the extremities?
6. Do you suffer from reduced/increased sweating?
7. Do you suffer from diarrhea?
8. Do you suffer from constipation?
9. Do you suffer from nausea or vomiting when eating?
10. Have you noticed any weight loss?
11. Do you suffer from erectile dysfunction (rated as “no” for female patients)?
12. Are/were there similar complaints in your family that have also been diagnosed by a doctor (tingling/numbness/weakness/pain; heart complaints)?
Bedside tests at the border zone area
1. 22 °C metal cube: Please indicate whether this was a cold or warm sensation or whether you did not feel any change in temperature
2. Pressure algometer at 4 mL: Was this pressure stimulus painful for you?

Note that the AmyloScan^®^ was designed in German. There is no validation for the English version

As the ultimate aim of the present study was to create a screening tool for ATTRv amyloidosis, both control cohorts (CIDP and PNP) were combined for the calculation of the final AmyloScan^®^. The discriminant analysis resulted in a high separation between groups, which was stable in the LOO analysis (Table 3).

**Table 3:** Predicted group membership following the discriminant analysis and Leave-one-out (LOO) analysis

			Predicted group membership		Total
			ATTRv amyloidosis	PNP	
Original	Count	ATTRv amyloidosis	20	1	21
		PNP	5	56	61
	%	ATTRv amyloidosis	95.2	4.8	100
		PNP	8.2	91.8	100
LOO	Count	ATTRv amyloidosis	20	1	21
		PNP	8	53	61
	%	ATTRv amyloidosis	95.2	4.8	100
		PNP	13.1	86.9	100

The discriminant function resulted in the specific coefficients, from which the simple function was derived (Table 4).

**Table 4:** Coefficients of the discriminant and simple function

	Discriminant function	Simple function	
		Answer	Point
1. Rapidly progressive course of the symptoms since the onset of the disease	0.260	Yes	1
2. Carpal tunnel syndrome?	0.526	Yes	1
3. Cardiac dysfunction?	0.225	Yes	1
4. Ocular dysfunction?	0.150	Yes	1
5. Muscle atrophia?	0.166	Yes	1
6. Hypo-/Hyperhidrosis?	0.016	Yes	1
7. Diarrhea?	0.313	Yes	1
8. Constipation?	0.005	Yes	1
9. Nausea/Vomiting?	0.176	Yes	1
10. Weight loss?	0.104	Yes	1
11. Erectile dysfunction?	0.080	Yes	1
12. Positive family history	0.557	Yes	1
1. 22 °C Metal cube: temperature change felt?	− 0.116	No	1
2. Pressure algometer at 4 mL: pain?	0.167	Yes	1

**Fig. 5:**
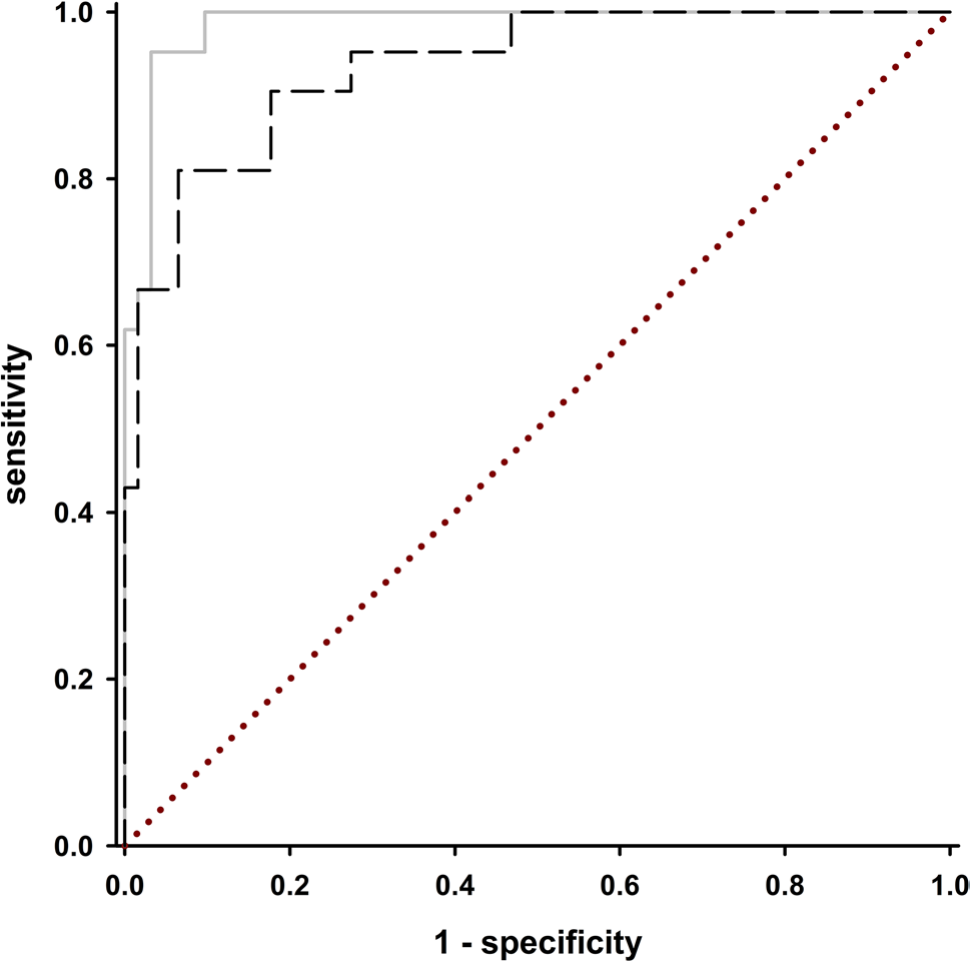
ROC curves for the final AmyloScan^®^ total score. Light gray curve = discriminant function; dashed black curve = simple function; red dotted curve = reference line

The ROC analysis revealed similar performance of the discriminant function (ROC AUC: 0.985, 95% CI: 0.966–1.000) compared to the simple function (only yes/no answers, additive score) (ROC AUC: 0.951, 95% CI: 0.904–0.998; Fig. 5) with confidence intervals overlapping. Due to its practical advantages of easy calculation and likely higher external validity, the simple function was chosen.

This resulted in a simple score with a scale of 0 to 14 points. As the Youden index did not reveal a single best separating value, we determined two thresholds. A value of 4 or above indicates an increased chance of amyloidosis (sensitivity: 95.2%, specificity: 72.6%). A value of 6 or above indicates a high chance of amyloidosis (sensitivity: 81.0%, specificity: 93.5%).

## Discussion

Hereditary transthyretin amyloidosis is a progressive genetic disease with an estimated mid-global prevalence of 10,186 persons, which fulfills the criteria for a rare disease [3]. However, the disease is underdiagnosed due to its unknown nature and heterogeneity; therefore, the prevalence might be much higher. To enable early diagnosis, it is necessary to be aware of characteristic symptoms and signs of the disease. Red flags for ATTRv polyneuropathy have been already published by experts [16]. However, to date, there is no easy-to-use screening tool that specifically checks for characteristic symptoms or signs and can be used to determine the probability of the presence of ATTRv amyloidosis. The successful implementation of such a screening tool is demonstrated by the example of the FabryScan, a tool consisting of a short questionnaire and three simple bedside test that has been shown to have good discriminative value for identification of patients with Fabry disease in patients with chronic extremity pain [17].

In the present study, we identified characteristics of ATTRv polyneuropathy by detailed investigation in a first step and developed the final AmyloScan^®^ in a second step. We were able to show that ATTRv polyneuropathy is characterized by specific symptoms and signs that distinguish it from polyneuropathies of other origins (e.g., CIDP and PNP of other causes) with high sensitivity and specificity. The AmyloScan^®^ consisting of only 12 questions and two bedside tests is quick to carry out and easy to score (only yes/no answer).

### ***AmyloScan***^***®***^***: characteristic questions***

Of the 15 questions that were part of the first AmyloScan® version, 12 were selected for the final version (Supplement Table 4). The first question on disease progression is not surprising, as ATTRv amyloidosis is known to be a rapidly progressing disease. Unlike diabPNP, for example, polyneuropathy due to ATTRv amyloidosis progresses rapidly and, if left untreated, quickly leads to a significant reduction in walking ability, immobility, and death [4]. It should be noted that in the first step of the study, we asked about the current course of the disease, i.e., under the respective treatment. As a result, more than half of the ATTRv patients described their disease as stable, while most of the CIDP patients and diabPNP reported a progressive course of disease. In order to rule out any bias due to the treatment, the question was rephrased and instead asked in the second step of the study about the overall course of the disease since the onset of symptoms.

The last question regarding a positive family history is also not surprising, as ATTRv amyloidosis is a genetic disease with autosomal dominant inheritance. As we found when conducting the first step of our study, signs of genetic manifestation can also go undetected in many cases (in non-endemic regions), as the disease may have gone undiagnosed in older generations due to a lack of awareness. It is known that the absence of a positive family history is a common reason for misdiagnosis [18]. To lose as few patients as possible due to the false assumption of a negative family history, in the second step we asked about possible neurological and cardiac symptoms of ATTRv amyloidosis in family members (tingling/numbness/weakness/pain; heart complaints) and not about a known diagnosis of ATTRv amyloidosis.

Three questions target possible other organ manifestations, i.e., carpal tunnel syndrome, ocular, and cardiac. Carpal tunnel syndrome is known to be a frequent manifestation of ATTRv amyloidosis. In a retrospective study, 56% of patients with ATTRv amyloidosis and even 13% of asymptomatic gene carriers were found to have a history of CTS, i.e., it can also precede a systematic manifestation and can therefore be regarded as an early red flag [19]. In the present study, the frequency of CTS was even higher (70%). While the heart is known to be one of the main organ manifestations, the eyes are affected somewhat less frequently, with ocular manifestations being described for certain mutations in particular [20]. In the present study, half of the ATTRv amyloidosis patients reported an ocular dysfunction such as blurred vision (not age-related), compared with only 26%/14% in the cohort of CIDP/PNP patients. These data are supported by the literature: Overall, 20% of patients were reported to have a history of glaucoma and/or vitreous opacities and up to 70% of patients report dry eyes [16], emphasizing the importance of ocular disturbance as a characteristic symptom, especially in combination with other organ manifestations.

The remaining seven questions of the AmyloScan^®^ target polyneuropathy symptoms (six questions for autonomic neuropathy, one question for motor neuropathy). Clinical presentation of ATTRv polyneuropathy differs depending on whether it is an endemic region or, as in the case of the country where the present study was conducted, a non-endemic region. In the latter, several phenotypes have been described: a small fiber PNP, a length-dependent all-fiber PNP mimicking CIDP, a multifocal PNP with onset in the upper limbs, an ataxic neuropathy, and an exclusively motor neuropathy [11]. As it seems difficult to capture this heterogeneity with a single questionnaire, we focused on symptoms that were reported by the vast majority of patients. In the present study, 80% of patients with ATTRv polyneuropathy reported at least one symptom of autonomic polyneuropathy, with erectile dysfunction, orthostatic dizziness and diarrhea being the most common symptoms. These results are consistent with data from the literature [21]. Autonomic dysfunction not only occurs frequently but also early in the course of the disease [22] and is therefore a clear red flag of ATTR amyloidosis. However, our data also show that only certain autonomic symptoms distinguish ATTRv polyneuropathy from polyneuropathy of other causes (i.e., dyshidrosis, diarrhea, constipation, nausea/vomiting, weight loss, erectile dysfunction) while others, such as orthostatic dizziness, are common in ATTRv amyloidosis but do not seem to be a distinguishing feature. Symptoms of motor polyneuropathy can also occur early in the course of the disease, which distinguishes ATTRv polyneuropathy from other neuropathies such as diabPNP [4]. Results from the clinical examination of the first study step were consistent with these findings, i.e., muscle weakness of the upper and lower limbs, atrophy and gait impairment were shown to be more frequent in ATTRv amyloidosis compared with diabPNP and/or CIDP. However, when trying to translate these signs into questions, the differences were partially lost. Consequently, only the item “muscle atrophy” was included in the AmyloScan® as a characteristic distinguishing feature.

Sensory symptoms and signs were common in patients with ATTRv amyloidosis, i.e., in step 1, all patients reported at least one sensory symptom in the lower limb, most commonly numbness and/or pain, and in the majority of cases also in the upper limb. Although the clinical examination showed significant differences between ATTRv amyloidosis and diabPNP with regard to sensory loss in the upper limb (70% vs. 26.7%), these could not be captured by history questions. CIDP is one of the most common misdiagnoses of ATTRv amyloidosis [23]. A study by Lorezon et al. investigated possible misleading features and possible distinguishing features of CIDP and late-onset demyelinating ATTRv amyloidosis [24]. In addition to autonomic dysfunction, loss of small sensory fibers above the wrists, upper limb weakness, and absence of ataxia, pain was a characteristic distinguishing feature, i.e., only 11% of CIDP patients but 59% of ATTRv amyloidosis patients suffered from pain. In line with this, more ATTRv amyloidosis patients reported pain than CIDP patients in the first step of the present study. Despite the lack of significance, we decided to include this question in the validation step and in particular to ask about burning pain, as the painDETECT questionnaire had shown a corresponding trend (data not shown here). Contrary to our original expectation, neuropathic pain was reported more frequently by CIDP patients than by ATTRv amyloidosis patients in the second step of the study. A decisive reason for this could have been the imprecise wording of the question (do you suffer from pain in your hands and/or feet (nerve pain)?). Other reasons could be the small patient cohort or the advanced stage of ATTRv amyloidosis with loss of function of the nerve fibers and the associated less frequent positive symptoms. Therefore, we decided to exclude this question from the final analysis.

### ***AmyloScan***^***®***^***: characteristic bedside parameters***

In the first item selection step, patients underwent a detailed sensory examination. In addition to the standardized DFNS-QST protocol [13], a validated bedside QST protocol was used to ensure the practicability of the screening tool [14, 15]. The results of the first step were validated in the second step with finally identification of two bedside sensory testing parameters that are characteristic of ATTRv polyneuropathy compared with polyneuropathies of other causes: hypoesthesia to cold and hyperalgesia to pressure, both performed in the most proximal area that is still clinically affected (border zone area) (Supplement Fig. 1). Hypoesthesia to cold reflects function of small Aδ-fibers, which can be impaired early in the course of ATTRv amyloidosis [1]. Hyperalgesia to pressure is considered a parameter reflecting central sensitization, i.e., an “increased responsiveness of nociceptive neurons in the central nervous system to their normal or subthreshold afferent input” [25]. Interestingly, polyneuropathies of other cause are rarely characterized by this underlying pathomechanism and rather by deafferentation [26]. Our results are partly consistent with a study by Escolano-Lozano et al., in which 24 patients with ATTRv polyneuropathy were compared with 48 patients with diabPNP using the QST protocol of the DFNS on the dorsum of one hand and foot [27]. While the authors also identified a significant loss of function with regard to cold sensitivity in ATTRv polyneuropathy, there were no differences in relation to pressure pain hyperalgesia. However, patients with ATTRv polyneuropathy were characterized by mechanical hyperalgesia, which is also considered a parameter indicating central sensitization.

### Strengths and weaknesses

As a strength, the AmyloScan^®^ was developed following a well-planned study procedure with a detailed examination in the first step and validation in the second step. The aim of the detailed examination was to characterize the patients as precisely as possible and thus identify previously unknown red flags. Despite the large number of items, we succeeded in developing an instrument that contains a short and simple questionnaire for the patients and two easy-to-understand bedside tests for the examiner, is easy to use, and can be carried out quickly. We decided to use simple yes/no answers instead of graded ones to guarantee an easy applicability with simple scoring. As the ROC analysis revealed similarly strong results between the discriminant function and the simple scoring, we assume that the simple scoring will also have higher external validity as it is less prone to overfitting to the current dataset.

Limitations of this study mainly relate to the study cohort. In the first step of the study, we focused on diabPNP and CIDP as the comparison cohort, as the former is the most common cause of polyneuropathies in the Western world and the latter is the most common misdiagnosis of ATTRv polyneuropathy. However, by focusing on these two groups, it is conceivable that characteristic distinguishing features have been either overestimated or not identified. Therefore, in the second step, we decided to also include polyneuropathies of other causes. The fact that results could also be confirmed in comparison to a more heterogeneous group of polyneuropathies speaks in favor of the AmyloScan®. Our aim was to develop a screening tool, i.e., focusing on early symptoms of ATTRv amyloidosis. In the present study, we included patients with confirmed ATTRv amyloidosis. As this is a rapidly progressing disease, many of these patients already had a pronounced symptom burden. However, to overcome this limitation and detect early signs of the disease, we (1) explicitly asked about initial symptoms in the medical history and (2) performed sensory testing in a mildly affected area (border zone area). In addition, most patients were in Coutinho stage 1 of the disease. Nevertheless, there is residual uncertainty regarding the significance of the characteristics identified here as early symptoms/signs. The small study cohort also limits the significance of our study. However, in order to achieve a certain significance despite this small group size, the patients were examined in detail in the first step of the study to identify also subclinical differences, and these results were further validated in the second step. Further limitations are the lack of analysis of the test–retest reliability, which is necessary in the further validation process, and the lack of blinding of the investigator regarding the diagnosis. Finally, the AmyloScan^®^ is especially designed for general practitioners in everyday clinical practice to support the suspicion of the presence of ATTRv amyloidosis and to identify patients at risk. It can never replace further diagnostics, including genetic testing, but can rather be regarded as a supporting tool for initiating further diagnostic steps in everyday clinical practice. Due to its simple and fast application, the AmyloScan^®^ could also help generate real-world data for research purposes.

## Conclusion

The AmyloScan^®^ is a simple screening tool consisting of a 12-item questionnaire and two easy-to-perform bedside tests, with a 14-point total score and two cut-off thresholds to indicate the likelihood of ATTRv amyloidosis. The AmyloScan^®^ has a good discriminative value to distinguish patients with ATTRv polyneuropathy from patients with polyneuropathy of other causes and can therefore be used as a simple screening tool in the early detection of the disease. Given the considerable clinical heterogeneity of the disease, the AmyloScan^®^ must be validated in larger cohorts to definitely confirm its usefulness in clinical practice.

## Supplementary Information

Below is the link to the electronic supplementary material.

Supplementary file1 (DOCX 25 KB)

Supplementary file2 (JPG 637 KB)

## Data Availability

The data presented here can be requested from the authors.
