# Supplementary material for: A new screening tool for early recognition of ATTRv polyneuropathy in clinical practice: AmyloScan®
Source: J Neurol. 2025 Sep 4;272(9):608. doi: 10.1007/s00415-025-13338-z (PMC12408649; doi:10.1007/s00415-025-13338-z)
Supplement: Supplementary file 1 — Supplementary file1 (DOCX 25 KB) [file 415_2025_13338_MOESM1_ESM.docx]

**Supplement material**

**Suppl. Table 1. Anonymized questionnaire (translated into English)**

| 1. When were you diagnosed with the disease (date of initial diagnosis)? |
| --- |
| 1. Looking back, what were the first symptoms you noticed? Please describe them as precisely as possible, including their location and time of first occurrence. |
| Current symptoms |
| 1. Do you suffer from changes in skin perception? (pain, tingling, burning, numbness, etc.)   If yes, please describe your symptoms in as much detail as possible. Please also state the exact localization of the symptoms. |
| 1. Do you suffer from muscular complaints? (muscle weakness, etc.)   If yes, please describe your symptoms in as much detail as possible. Please also state the exact localization of the symptoms. |
| 1. Do you suffer from complaints of the cardiovascular system? (palpitations, cardiac arrhythmia, shortness of breath, etc.)   If yes, please describe your symptoms in as much detail as possible |
| 1. Do you suffer from kidney problems? (increased urination, etc.)   If yes, please describe your symptoms in as much detail as possible |
| 1. Do you suffer from gastrointestinal complaints? (diarrhea/constipation, nausea, etc.)   If yes, please describe your symptoms in as much detail as possible |
| 1. Do you suffer from any complaints that affect the brain? (thinking/concentration problems, etc.)   If yes, please describe your symptoms in as much detail as possible |
| 1. Do you suffer from any other complaints? (dizziness, changes in sweating, weight loss, etc.)   If yes, please describe your symptoms in as much detail as possible |
| 1. Are there similar complaints in your family?   If yes, please describe your symptoms in as much detail as possible. Please also state who is/was affected (parents, siblings, etc.) and at what age the symptoms first appeared. |

**Suppl. Table 2. Characteristics of patients with ATTRv amyloidosis examined in the first step of the study (n=10)**

| **Male, n (%)** | 7 (70) |
| --- | --- |
| **Age at examination, mean ± SD (range)** | 58.5 ± 10.0 (40-73) |
| **Symptom duration, years (range)** | 8.8 ± 8.3 (2-28) |
| **Gene mutation, n (%)** |  |
| Val30Met | 5 (50) |
| Ile104Asn | 2 (20) |
| Arg54Thr | 1 (10) |
| C69+5C>C | 1 (10) |
| Glu109Gln | 1 (10) |
| **Coutinho stage, n (%)** |  |
| 0 | 0 |
| 1 | 7 (70) |
| 1-2 | 1 (10) |
| 2 | 2 (20) |
| **PND score, n (%)** |  |
| 0 | 0 |
| 1 | 5 (50) |
| 1-2 | 1 (10) |
| 2 | 1 (10) |
| 2-3 | 1 (10) |
| 3b | 2 (20) |
| **Disease modifying drug, n (%)** |  |
| Tafamidis | 5 (50) |
| Patisiran | 5 (50) |
| SD, standard deviation  PND, polyneuropathy disability score | |

**Supp. Table 3. Items of the AmyloScan® 1.0**

| **Questions** | | **Response options** |
| --- | --- | --- |
| 1. | Symptom duration | Years |
| 2. | Total course of the symptoms since the onset of the disease | Stable, decreasing/improving, slowly progressive/worsening, rapidly progressive/worsening |
| 3. | Course of the symptoms over the last 6 months | Stable, decreasing/improving, slowly progressive/worsening, rapidly progressive/worsening |
| 4. | Diagnosis or symptoms of a carpal tunnel syndrome (i.e., pain dysesthesia thumb to ring finger) | Yes/no |
| 5. | Symptoms of cardiac dysfunction (e.g., dyspnea, palpitation, cardiac arrythmia, pre-existing cardiac diseases) | Yes/no |
| 6. | Symptoms of ocular dysfunction (e.g., blurred vision) | Yes/no |
| 7. | Muscle atrophy | Yes/no |
| 8. | Restless legs syndrome | Yes/no |
| 9. | Uncertainty when walking | Yes/no |
| 10. | Slow gait | Yes/no |
| 11a. | Hypo-/Hyperhidrosis | Yes/no |
| 11b. | Diarrhea | Yes/no |
| 11c. | Constipation | Yes/no |
| 11d. | Nausea/Vomiting | Yes/no |
| 11e. | Orthostatic dizziness | Yes/no |
| 11f. | Weight loss | Yes/no |
| 11g. | Incontinence | Yes/no |
| 11h. | Erectile dysfunction | Yes/no |
| 12. | Neuropathic pain | Yes/no |
| 12a. | Neuropathic pain intensity | 0-10 (no pain, worst imaginable pain) |
| 12b. | Neuropathic pain course | Stable, improving, worsening |
| 13. | Tingling | Yes/no |
| 13a. | Tingling intensity | 0-10 (no tingling, worst imaginable tingling) |
| 14. | Positive family history (similar symptoms e.g. tingling, numbness, weakness, pain, cardiac complaints) | Yes/no |
| 15. | (Mis-) Diagnosis CIDP | Yes/no |
| 15a. | Improvement of symptoms following CIDP therapy | Yes/no |
| **Bedside tools: Dorsum of the hand** | | **Parameters** |
| 1. | TipTherm | Number of stimuli recognized as cold (x/4) |
| 2. | Neuropen Neurotip | Blunt touch/pinprick |
|  |  | Pain intensity 0-10 (no pain, worst imaginable pain) |
| 3. | Bedside algometer | 4mL painful: Yes/no |
|  |  | 4mL pain intensity 0-10 (no pain, worst imaginable pain) |
|  |  | Pressure pain threshold mL |
| 4. | Tuning fork | Vibration detection threshold |
| **Bedside tools: Border zone area** | | **Parameters** |
| 1. | 22°C metal cube | Cold/warm/no temperature change felt |
|  |  | Cold intensity 0-10 (no coldness, strongest imaginable coldness) |
| 2. | Bedside algometer | 4mL painful: Yes/no |
|  |  | 4mL pain intensity 0-10 (no pain, worst imaginable pain) |
|  |  | Pressure pain threshold mL |

**Suppl. Table 4. Questions of the original AmyloScan® German version**

| 1. Haben sich Ihre Beschwerden seit Beginn der Symptome rasch verschlechtert/zugenommen? | Ja/nein |
| --- | --- |
| 1. Haben/hatten Sie aktuell oder in Ihrer Vergangenheit ein Karpaltunnelsyndrom oder Symptome eines solchen (Schmerzen/Missempfindungen Daumen bis Ringfinger)? | Ja/nein |
| 1. Haben Sie Herzbeschwerden wie Luftnot, Herzrasen, Herzrhythmusstörungen oder andere Herzvorerkrankungen? | Ja/nein |
| 1. Haben Sie Sehstörungen wie Verschwommensehen (nicht im Rahmen einer Altersfehlsichtigkeit)? | Ja/nein |
| 1. Leiden Sie an einem Muskelschwund im Bereich der Extremitäten? | Ja/nein |
| 1. Leiden Sie an vermindertem/vermehrtem Schwitzen? | Ja/nein |
| 1. Leiden Sie an Durchfall? | Ja/nein |
| 1. Leiden Sie an Verstopfung? | Ja/nein |
| 1. Leiden Sie an Übelkeit oder Erbrechen beim Essen? | Ja/nein |
| 1. Haben Sie einen Gewichtsverlust bemerkt? | Ja/nein |
| 1. Leiden Sie an einer Erektionsstörung (nur für männliche Patienten)? | Ja/nein |
| 1. Gibt/gab es in Ihrer Familie ähnliche Beschwerden, die auch ärztlich diagnostiziert worden sind (Kribbeln/Taubheit/Schwäche/Schmerzen; Herzbeschwerden)? | Ja/nein |

**Suppl. Figure 1. Bedside devices. Displayed are the devices used for the final AmyloScan®. (1) 2.7 × 2.7 × 2.7-cm metal cube for thermal perception/pain. The metal cube is applied for three seconds to the skin. The patient is asked about the quality of the stimulus, i.e. cold/warm perceived or not perceived. (2) 10-mL syringe for pressure pain sensitivity. The bedside algometer (10-mL syringe sealed with a plug and felt with a contact area of 1cm) is placed above a muscle in the border zone area (arch of the foot when testing the foot, Musculus gastrocnemius when testing the lower leg, Musculus vastus medialis when testing the upper leg). The syringe is slowly compressed with constant speed (1ml per second) up to 4mL. The patient is asked whether this compression was painful (yes/no).**
