# Supplementary figures and images for: A new screening tool for early recognition of ATTRv polyneuropathy in clinical practice: AmyloScan®
Source: J Neurol. 2025 Sep 4;272(9):608. doi: 10.1007/s00415-025-13338-z (PMC12408649; doi:10.1007/s00415-025-13338-z)

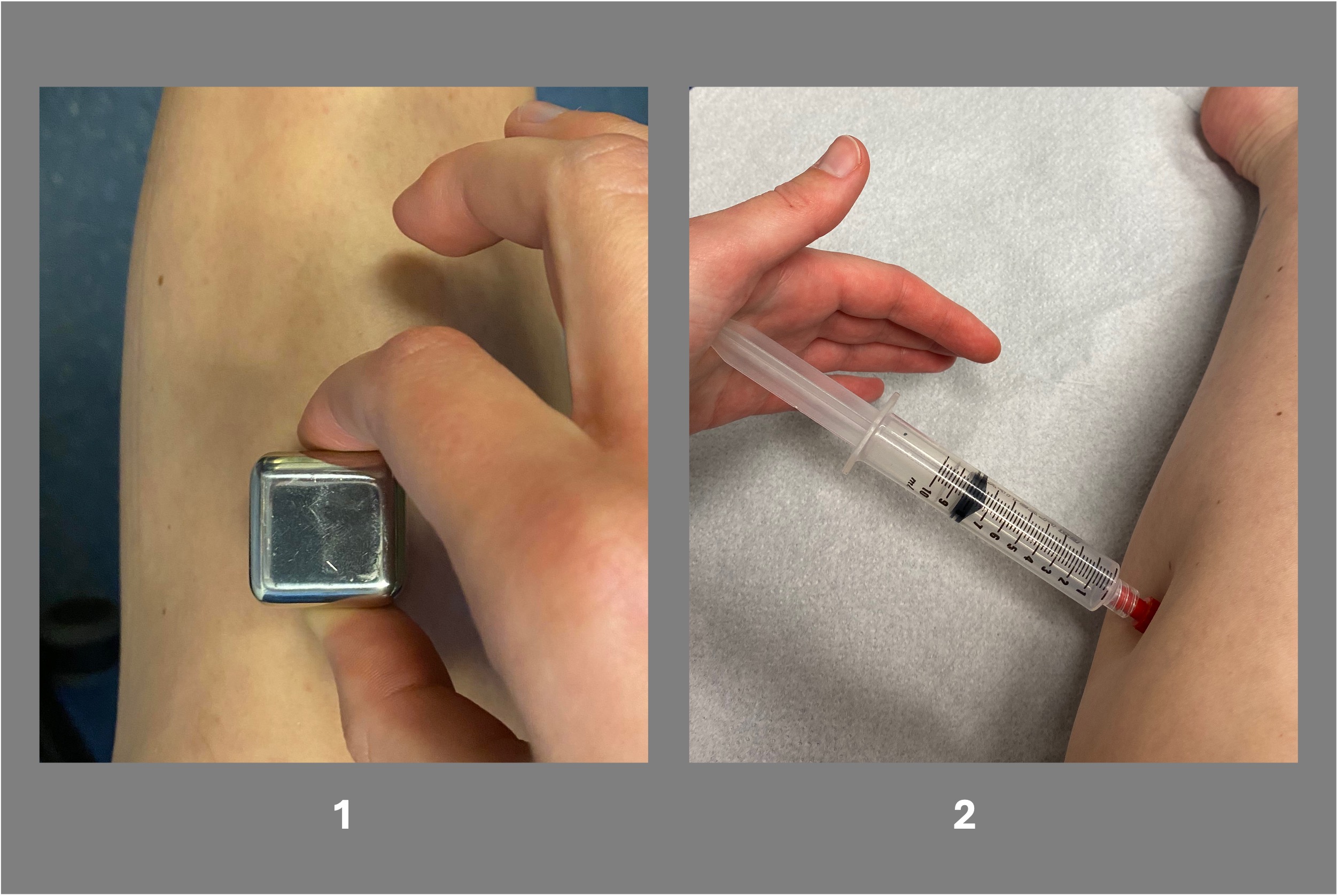

Supplement: Supplementary file 2 — Supplementary file2 (JPG 637 KB) [file 415_2025_13338_MOESM2_ESM.jpg]
